# Supplementary material for: Are carbon emissions trading and green financial instruments synergistic? -Comprehensive quantitative research based on content analysis
Source: PLoS One. 2024 Mar 7;19(3):e0298601. doi: 10.1371/journal.pone.0298601 (PMC10919601; doi:10.1371/journal.pone.0298601)
Supplement: S1 Data — (ZIP) [file pone.0298601.s001.zip › Data/地级市Z_F工作报告.zip!/║┌┴·╜¡/╥┴┤║╩╨/└┤╫╘╣1⁄2╓┌║┼í╛┬φ┐╦╩2╛▌═°í┐_2014.docx]

各位代表：

现在，我代表市政府，向大会报告工作，请予审议，并请市政协委员和其他列席会议的同志提出意见。

一、2013年工作回顾

刚刚过去的2013年，是我市三次创业、转型跨越取得突破性进展的一年。一年来，我们在市委的坚强领导和市人大、市政协的有力监督支持下，按照市委提出的瞄准六个方向、打好五个硬仗的工作要求，紧紧依靠全市广大干部群众，真抓实干，合力攻坚，全市经济社会总体向好的局面进一步巩固。

(一)经济保持较快增长。预计，全年地区生产总值完成286亿元，同比增长10%。在嘉荫县遭受百年不遇洪灾的严峻考验下，全市粮食总产达到 97万吨，与上年持平；农业增加值实现133.2亿元，增长14.5%。规模以上工业实现增加值46亿元，增长13%。全社会固定资产投资283.5亿元，增长30%。社会消费品零售总额实现86.5亿元，增长13%。全市城镇居民人均可支配收入和农民人均纯收入分别增长13%和9.8%。全口径财政收入35.1亿元，增长16.2%，公共预算收入增幅位居全省第二位。

(二)产业项目和园区建设取得重大突破。全年新建续建产业项目388个，完成投资127亿元，占固定资产投资比重由上年的42%提高到45%。鹿鸣钼矿、西钢高速线材、新能源汽车等15个重大产业项目建成投产，佳龙精品酒店主体完工，汇源绿谷项目全面启动。园区建设加速推进，铁力工业园区落地项目 26个，累计投资33亿元。高速列车制动盘、耐磨材料等10个项目试投产，新增产值2亿元、利税1430万元，两年时间，就建起了一座工业新城，成为全省园区建设的典范。嘉荫跨境(木业)园区晋升为省级工业示范基地。翠峦绿色食品加工园区通过政策引导和扶持，签约入驻小微企业40户。截至目前，八个园区累计入驻企业121户。全年“3+X”产业实现增加值123亿元，增长11.7%，占GDP比重达到43%。

(三)生态功能区建设深入推进。全面实施了《大小兴安岭林区生态保护与经济转型规划》和天保二期工程，主动停止了天然林商品性采伐，全年更新造林97万亩，森林抚育314万亩，首次实现全年无森林火灾。查处各类林政案件267起，依法收回林地4390亩。持久开展保护红松行动，荣获“中国红松之乡”称号。成立了保护白头鹤联合会，珍贵物种保护领域进一步拓展。争取节能减排、土地整理项目35个，汤旺河、新青、美溪、西林、金山屯污水处理厂和伊春区垃圾处理厂建成投用，全市集中饮用水源地水质、二级以上空气质量达标率均达到100%。万元GDP综合能耗下降3.2%。成功举办了第三届东北亚(伊春)生态论坛、“伊春现象”北京论坛暨“美丽中国·绿色伊春”环保公益发布会、“驻华大使夫人伊春行”等重要活动，伊春生态文明建设的影响力得到了有效增强。

(四)城市功能品位整体提升。全面启动实施城市提档升级工程，铁金、浩南公路建设加速推进，新建改造回龙湾大街、友好滨水路、乌马河通府路等20条道路，“三横五纵”路网进一步完善。省运会体育场馆主体工程全面竣工。“三供两治”力度加大，开工建设了中心城区燃气工程，华能热电联产项目继续挑战北方冬季施工极限，中水管线工程在零下30度严寒中仍在紧张施工。全市供热、供水普及率达到 73.1%和76.5%，分别提高4个和2个百分点。林都机场、高速路入城口、伊春河两岸、水上公园、步行街等重点工程陆续完工。对中心城区主街路和重要节点的临街建筑进行了节能保温整饰，对市中心区16条道路及12个公园、广场实施了改造升级，全市城区绿化覆盖率达到 56.5%。“创文”取得阶段性成果，获全省文明城市贡献奖。结合拆违，实施了大规模的拆墙透绿透景工程，使城市更加通透舒朗，“生态为魂、欧典为型”的建筑风格进一步彰显。

(五)改革开放进一步深化。国有林改取得新进展，启动了活立木交易市场，全年流转林地7271公顷、交易额5064万元。森林经营增汇减排项目试点取得突破，在国内完成了第一笔碳汇交易。中国林权交易所伊春交易中心正式签约。简政放权，取消行政审批许可71项。强化财政预算管理，“三公”经费同比压缩 15%。社会管理不断创新，取消街道层级，在全省率先实现了扁平化管理。对外开放空间不断拓展，积极组织参加了亚布力中国企业家年会、东北东部城市12+2论坛、上海浦东产业项目推介恳谈会等重大对外交流活动，与罗马尼亚阿祖加市缔结友好城市。

(六)民生持续改善。全市地方财政用于民生支出53亿元，增长23.8%，20件民生实事完成19件，1件正在推进。各地紧盯棚改完成时限，克服重重困难，纷纷自我加压，棚改攻坚战取得重要进展，铁力市、嘉荫县、金山屯、乌伊岭、翠峦、红星、西林消灭了区局址棚户区。全年开工建设保障性住房6.23万套、344万平方米，惠及人口16万。为市直机关、事业单位在职职工年人均增加津补贴3600元、一次性奖金2050元、住房公积金匹配比例由5%提高到10%。临时用工年人均增资3600元。企业退休人员养老金年人均提高 3060元。高考成绩实现八连升，理科平均分排名跃居全省第 4位。投入7700万元，为县区增加了急需医疗设备。新建改造300平方米以上社区10个。投用的新能源公交汽车和公共自行车运营系统受到市民普遍欢迎。创作编排了旅游晚会《兴安四季情》，推出了《山里女人山里汉》、《不败雄心》等反映小兴安岭地域文化的影视作品。在国际国内大型比赛上获金牌54枚，50万人次参与全民健身运动。平安伊春建设取得重大成果，夺得龙江首个全国社会管理综合治理最高荣誉———“长安杯”。

此外，民族宗教、外事侨务、双拥共建、人口计生、妇女儿童等各项工作都取得了新进展。

各位代表，这些成绩的取得，凝聚着全市上下的共同努力。在此，我代表市政府向全市各族人民和社会各界表示衷心的感谢！

回顾过去的一年，我们既为推进了伊春各项事业的进步而欣慰，同时也为发展中存在的问题和不足而自省。主要是：体制机制束缚依然较多，深水区的改革任重道远；区域经济发展不平衡，一些区局缺少增加财源和带动职工就业增收的好项目，提高城乡居民收入的任务艰巨；城市累积负债增多，融资难度不断加大；民生保障能力还不强，市民反映强烈的喝干净水、吃放心菜、住暖屋子等诉求还有待下大气力解决；政府职能转变和发展环境仍不适应加快发展的要求。对这些问题，我们要实事求是，深入分析，找准对策，一如既往地努力攻坚！

二、2014年主要工作

今年政府工作的总要求是：坚持以党的十八大和十八届三中全会精神为指导，认真贯彻落实省、市委全会和经济工作会议部署，牢牢把握“改革创新、稳中求进、进中求好”主基调，以强市富民为核心，深入推进三次创业、转型跨越，促进经济持续健康发展，保持社会和谐稳定，加快建设美丽富庶、文明和谐、健康幸福新伊春。主要任务是：统筹做好改革、发展、生态、民生四篇文章，重点抓好创建全国文明城市、举办第十三届省运会和第三届中国(伊春)国际森林产品博览会三件大事，努力办好十件民生实事。

根据市委全会对今年形势的分析判断，综合考虑总量基数、潜在增长率和不确定性因素，初步安排 2014年全市地区生产总值、规模以上工业增加值、公共预算收入分别增长10%，固定资产投资增长26%，社会消费品零售总额增长14%，城镇居民人均可支配收入和农民人均纯收入分别增长10%和13%。为实现上述目标，突出抓好四项工作：

(一)稳步推进改革。把改革创新贯穿于经济社会发展的各个领域，从当前最急迫、百姓最期盼、制约发展最突出的问题改起，以改革的主动赢得发展的主动。

推进机构改革。针对停伐实际，对林管局(林业局)木材生产、机械、经销等部门调整优化。加快推进卫生、人口计生、食品药品监管等部门整合。理顺城管、住建、规划等部门职责关系，解决多头执法问题。摸清事业单位底数，年底前完成分类。理清各区局职能、机构、人员等情况，为分步解决政社、政企分离做好基础工作。

推进行政审批制度改革。落实国家和省取消下放的行政审批事项，该放的坚决放到位，确需保留的向社会公示。能够进驻中心办理的审批事项，一律纳入中心。

推进林业综合改革。以建立国家林业综合改革试验区为方向，深化国有林权改革，放大试点综合效应。积极引进战略投资者，盘活民有林资源，充分释放林地生产力。推进林业资本化运作，争取纳入国家森林生态补偿和森林经营碳汇交易试点。借鉴吉林森工集团运作经验，率先在南四局探索集团化经营模式，组建多元化经营公司，做强森工实体经济。

推进投融资体制改革。组建市政公用集团，融通城市基础设施建设资金。适时启动供热、供水、公交市场化改革。成立文化旅游投资公司，通过抵押形式融资，为城市文化旅游发展提供资金保障。争取将农发行改为农林发展银行或增设“林业发展部”，将支持重点向林业倾斜。支持兴业小额贷款公司改制，逐步向村镇银行过渡。

推进财税体制改革。建立财源建设奖励基金，提高市对区增值税、营业税增收返还比例。建立生态功能区建设补助机制，加大市对区财政转移支付力度。理顺收入划分，将区级行政性收费、政府性基金中的市所得部分划为区级收入。细化预算编制，实行收支统筹、归口管理。加强预算约束，减少追加预算。健全债务风险防控制度，强化债务资金管理。适时启动公车改革。

推进教育、卫生、文化改革。创新办学体制机制，在幼儿园、小学、初中和高中学校中分别拿出1所进行试点，引入社会资本办学。引进战略投资者，对1-3个公立医院和养老院进行兼并、重组或收购，形成有效的市场竞争机制。完善文化管理体制，理顺部门与文化企事业单位的关系，搞活文艺院团的市场化经营。

(二)持续推动发展。结合林区现阶段实际，重点在产业升级、对外开放、科技创新、城市建设上实现新突破。

第一，加速提升产业竞争力。以存量带增量，放大资源优势，推进产业合作式、技术升级式、数量扩张式发展。

狠抓产业链延伸与扩大。矿产开发及冶金建材业，突出抓“钢钼金玉”四条产业链。钢产业链，按照绿色、低碳、循环发展要求，支持西钢技改，加快从钢材为主向钢产品延伸。钼产业链，以钼冶炼、钼化工、钼金属为重点，深度开发钼矿资源。金产业链，加强与深圳九五集团合作，推进金叶珠宝黄金制品加工项目落地，综合利用乌拉嘎金矿尾矿资源，延伸发展金制品。玉产业链，加大对桃山玉矿的保护和集约利用，引进战略投资者和技术，生产终端产品，打造桃山玉街，建设中国新玉乡。深化与省地矿局的战略合作，抓好秋雨山铅锌、东风硅石等项目探矿权的设立及招拍挂。森林食品突出抓“红蓝黑+林药+林畜”五条产业链。红松产业链，以汇源、中盟天隆为龙头，加速推进坚果精深加工生产线建设，争取在下一个红松籽丰产期到来前使加工能力达到2万吨以上，实现红松籽不出市。蓝莓产业链，以友好万亩蓝莓基地为依托，以越橘庄园、忠芝、双蓝等企业为带动，打造从繁育种植、加工销售到蓝莓文化的全产业链条。黑木耳产业链，大力开展食用菌产业素质升级行动，引进黑尊集团，建设总投资5亿元的黑木耳新技术产业化项目，提高食用菌生产加工能力和水平。林药产业链，加大对格润、葵花等制药龙头企业的扶持，形成拳头产品，带动林药集约化、规模化、自然化种植，做大上游环节。林畜产业链，以做大“伊春森林猪，全程可追溯”项目为示范，打开销售出口，以销带养，取得突破后将这一模式在其它养殖项目上推广。同时，围绕“七带一谷”建设，扩大林下经济规模，创新农业经营主体，完善“龙头+基地+农户”利益联结机制，年内新增专业合作社70个、经营大户50个以上。

推动旅游产业升级。借助国家5A级景区的优势，整合提升北部景区资源，合力做出大品牌。抓住铁金、浩南公路建成通车后形成的新优势，围绕“干”字型布局，集中打造金山小镇、回龙湾、仙翁山、带岭影视城等东南部旅游大环线(伊春—南岔—铁力—伊春)、小环线(伊春—南岔—带岭—乌马河)。以此为依托，深度开发周边客源市场，联合开发省外远程客源。把做强冬季旅游作为实现四季均衡的主攻方向，借鉴亚布力、张家口等地国际化运营模式，加快推动现有滑雪场提档升级，扩大全国职工滑雪邀请赛、伊春国际冰壶邀请赛等品牌活动的影响力。整合铁力和桃山滑雪、狩猎、温泉、水上乐园等资源和项目，设计新的线路和产品，在南部率先实现冬季游的重大突破。整合夏季节事展会活动，按照“国际”办会标准，通过市场化运作，集中打造“中国(伊春)国际森林产品博览会”新品牌。加强与国旅、中青旅等知名旅行社的合作，建立游客服务中心，大力发展智慧旅游，充分利用新媒体和自媒体开展旅游宣传推介，全力扩大市场份额。

本着“有进有出、优化发展”的原则，保大压小，淘汰落后产能，巩固发展家具、木制工艺品等传统优势产业，大力发展新材料、新能源、新技术以及时尚产业，做优做强“X”产业。

大力发展现代服务业。注重实体经济与市场的深度融合，抓住生产、消费和对俄贸易三条主线，加快建立综合配套物流产业体系。建立“伊春森林厨房”、“桃山市民农园”等电子商务平台。推进“宽带伊春”建设，支持各家电讯企业联手做大信息服务业。注重商业模式设计与推广，鼓励社会资本大力发展养老服务业。

启动新三年产业项目攻坚战。全年安排重大产业项目24个，总投资132亿元，年度投资38.4亿元。市级层面突出抓好总投资75亿元的“汇源绿谷”项目，按照建设“生态硅谷”的构想和“资源科学利用、产业自觉集成、品牌世界知名、驱动地方发展、引领生态文明”五大目标，搞好顶层设计，加速推进会展中心、企业家庄园、物流商贸中心、小浆果种植、特种养殖等项目建设。以此为示范，吸引更多一流企业、顶尖项目、高新技术向“绿谷”集聚，最终将其打造成世界知名的绿色产业集中地和代名词。

第二，全力提升对外开放层级。立足产业基础和资源优势，突出借力发展，做大外向型经济。

深入开展务实招商。围绕主产业链延伸、八个园区建设以及教育、医疗、养老、公益事业等领域改革，充分发挥10个市级专题招商工作组的作用，明确招引目标，突出招引重点，一对一、点对点招商，切实提高成功率。坚持外商内商一视同仁，给予本土企业家同等优惠政策。全年实现招商引资到位资金120亿元。

深度推进对俄合作。调动各种力量，合理分工，全力做好“中国—俄罗斯博览会”参展工作。加强通道建设，千方百计推动嘉荫口岸复关。在继续推进嘉荫跨境(木业)园区规划建设的基础上，采取分税制的合作方式，在绥芬河市开发建设总面积4.8平方公里的“中国伊春(绥芬河)木材储备加工交易园区”，整合森工企业闲置生产要素，成立伊春对俄林业开发集团，牵手搭靠强势边贸企业，借船过江，力争过境采伐和入境加工实现“双百万”。

积极争取政策支持。围绕“五大规划、一个工程”，研究政策，深挖潜力，争取在建立国家林业综合改革试验区、绿色发展示范区以及享受惠农政策等方面取得实质性突破。

第三，加快推进科技创新。把科技创新摆在转型跨越的核心位置，全力推动科技创新与经济社会发展的深度融合。

强化自主创新。重点围绕主产业链中的关键技术开展攻关，力争在全国率先出台蓝莓等种植加工行业技术标准。建设小兴安岭蓝莓博物馆，用文化引领森林食品产业发展。启动实施工信部“绿色伊春”区域品牌培育试点，大力研发新品、多出优品、打造精品，着力刻画代表城市形象的主体产品，提升“伊春制造”的品质和“伊春创造”的影响力。

释放科研机构活力。建立与贡献相匹配的股权激励、科技成果处置权、收益权自主支配等创新收益制度，激活各类科技研发资源。鼓励支持市林科院、带岭林科所及双丰农科所等科研机构积极与企业对接，通过“产学研用”协同创新，将先进的科技成果推广应用到产业提档升级中。依托省林下资源协同创新中心，深化与东林、东农的研发合作，用新技术、新成果推动产业向价值链中高端跃进。

实施人才兴安岭工程。把发挥人的创造力作为推动科技创新的核心，加大人才培养力度，使各类创新型人才脱颖而出。完善用人机制，按照有利于发挥科技人员积极性的要求，改进科研管理方式，鼓励人才自由流动和组合。出台更具吸引力的聚才纳智政策，推进落实万名大学生引进计划，吸引各类人才到伊春创新创业。健全人才向企业、基地等生产一线流动的激励机制，为基层科技创新“施肥增养”，促进增长模式由要素推动向创新驱动转变。

第四，打造北方宜居森林城镇群。依靠市场力量和规划引导，集中打造中心城区、铁力市、嘉荫县、南岔、新青、金山屯—西林“一主五副”的主城区和副城区，推动人口合理集聚。

做好棚改收官工作。集中抓好城镇周边、公路沿线棚户区拆迁工作，把该拆的拆到位。通过开发商让利与政策扶持，尽快消除现存棚户区，盘活积压房源。完善棚改小区综合服务功能，推广电力嘉园物业管理模式，依靠政府、市场和群众自治三方力量提升小区管理水平。

加强枢纽型和功能型基础设施建设。加快推进铁金、浩南公路建设，确保年内建成通车。抓好市公路客运中心和伊嘉公路改扩建前期手续报批，推进伊翠铁路改线工程，力争年内全部开工。改造升级回龙湾大街、青山大街等 10条市政道路，推动“三横五纵”路网整体升级。加快推进华能热电、兴安湖水库输水等项目建设，提高“三供两治”保障能力。强化防汛防洪能力建设，加快推进水毁和不达标堤防的修复。搞好村屯整合，建设美丽乡村。

突出特色搞好美化。注重城市绿化的园林设计，增加彩叶、阔叶树种比重，扩大成片绿地面积。对山河大道等重点景观带进行改造，“显山露水”，对部分山体实施亮化，提升城市立体空间感。

以“创文”推动城市建设管理升级。动员全市力量，全面落实各项目标要求，围绕城乡环境、市场秩序、市民文明素质等方面，突出重点，狠抓关键，补齐短板。集中抓好城市进出口道路、铁路沿线、集贸市场、背街小巷等薄弱区域环境综合治理，力保顺利通过中央文明委的全面检查验收。

(三)建设中国最好的生态功能区。以全面停止天然林商品性采伐为新起点，加大森林培育力度，促进森林资源质与量同步高效恢复，夯实绿色发展根基。

坚守生态保护红线。加强森林资源管护，重点对农林交错地带严格监管。强化“三防”，注重科技应用，提升森林防火、防盗和有害生物防治能力。出台湿地保护办法，促进湿地资源可持续利用。强化白头鹤、中华秋沙鸭等各类自然保护区建设，依法查处乱捕滥猎行为。认真研究建立国家公园制度的步骤、标准和机制，积极争取政策支持，逐步把伊春北部地区的14个保护区、9个森林公园、3个地质公园都纳入进去，整体规划建设“伊春国家公园”。

大力培育森林资源。抓好森林抚育补贴试点，精细化抚育森林，促进低质次生林向优质速生林转变，全年抚育中幼龄林311万亩。优化林分结构，恢复重建阔叶红松林，加大三大硬阔的补植更新力度，使树种组成比例分别达到20%和30%。着力强化对林间空地、线路走廊、矿山废弃地的植被恢复和利用。积极开展平榛等经济果林和胡枝子生物质能源林的改培营造。退耕还林13万亩。加强标准化苗圃建设，苗木产量达到1.5亿株。

深入推进节能减排。加快钢铁、化工建材等行业落后产能的淘汰和改造，逐步停用10吨以下供热燃煤锅炉，降低污染排放总量。培育生态教养，倡导绿色消费，减用一次性木制品，不食野生动物，推广使用新能源汽车，实施公交汽车“油改气”，扩大公共自行车项目覆盖面，创建生态文明建设先行示范区和绿色消费示范市。

(四)提高民生保障的均衡性、普惠性。坚持“问题导向”，将群众期盼作为民生工作的着力点、落脚点，力促社会公平正义。

狠抓创业增收。在巩固发展非公有制经济、职工自营经济、劳务经济的同时，认真研究促进增收的新模式。推广汤旺河区发展“居室经济”的做法。完善就业创业扶持政策，发挥大学生创业园等创业孵化基地的作用，扩大小额贷款规模。

提高社会保障水平。强化企业基本养老保险扩面征缴，着力扩大非公有制企业、个体劳动者和灵活就业人员的参保覆盖面。加快推进医疗保险和生育保险市级统筹，完善新农合制度。大力开展养老和孤残儿童救助，抓好养老机构和社会福利中心建设。健全住房公积金制度，年内实现21个县区全覆盖，并根据财力逐步提高匹配比例。

创新社会治理。放大社区扁平化管理效应，实行居民代表常任制，提高居民自治水平。落实信访属地化管理，推行网上受理制度，密切关注网上舆论和社会舆情，推动涉法涉诉信访纳入法治轨道解决。坚持生命至上，健全安全隐患排查和监管体系，遏制重特大安全事故发生。突出抓好食品药品质量监管，保障群众“舌尖上的安全”。健全应急管理救援机制，提高预防自然灾害和应对突发事件的能力。扩大“长安杯”成果，提升平安伊春建设水平。

大力发展社会事业。进一步改善办学条件，提高教育教学水平，学前三年毛入园率和义务教育标准化学校分别达到80%以上，本科二表以上入段率达到48%。弘扬“尊师重教”、“为人师表”传统美德，开展在职教师“知荣明耻，共抵三乱”行动。依托电大远程教育平台，建立社区终身教育体系。以“三甲”医院为载体，拓展与省内外著名医疗机构的合作，提升重大疾病救治能力。加快发展健康服务业和养老服务业，增加老年人服务窗口、活动场所，探索开发系列康体保健、养老养生产品。完善公共文化服务体系，挖掘森林文化内涵，培育发展创意、动漫等新兴文化业态，策划推出一批文艺新品。启动十三届省运会倒计时，调动各方力量，办一届精彩、节俭、成功的运动会，树立美丽伊春新形象。

本着量力而行、尽力而为的原则，重点办好十件民生实事，让发展成果普惠林区群众。

1、分步骤、梯次解决政企合一区(局)机关事业单位人员工资待遇偏低问题；通过争取政策，搞活经营，增加林业局职工收入。

2、投资10亿元，改造棚户区12330户，新建公租房1500套。

3、投资2.9亿元，完善“三供两治”工程，开工建设中心城以及南岔、乌伊岭、朗乡、双丰、桃山污水处理厂；对市中心区二水厂以及朝阳、普新、五二三、伊木加、站前小区共 121栋楼房6049户居民锈蚀自来水管道进行改造升级；新建青山大街、伊木加供水管线；对中心城区部分供热管网和分户供热进行改造；续建中心城区燃气工程。

4、抓好“菜篮子”工程，在铁力市新建300亩设施蔬菜生产基地，伊春区、友好、翠峦、乌马河各新建200亩。

5、投资1879万元，整体改造红旗、隆福、工联、永兴、华林、机修街6个旧小区；更换15台危旧变压器和老化线路；改造升级红旗、黎明、五六、中植村等10个旧小区宅前道路，新建15个停车场。

6、投资3000万元，建设市中小学生综合实践基地；免收90%中职学校学生学费，对20%普通高中学生给予助学补助，一次性资助考入本科二表以上院校的贫困学生1000名。

7、投资2000万元，为基层医疗机构购置医疗设备；投资2400万元，建设市全科医生临床培养基地和老年病医院；扩大医疗救助范围，将低收入群体中的重残、老年人和重度精神病人纳入救助范围。

8、新建、改扩建300—500平方米达标和标准型社区服务中心2个，1000平方米示范型社区服务中心3个。

9、增开伊春区至友好区的公交线路。

10、投资1897万元，购置现代化清雪设备35台；冬季为环卫工人每人每天增加御寒补贴10元；增设临时休息点；从今年起，环卫节组织机关干部义务劳动，环卫工人放假一天。

三、加强政府自身建设

把党的群众路线教育活动贯穿于作风建设的全过程，努力打造为民、务实、清廉政府。

各级政府工作人员要对党忠诚、对事业尽职、对人民负责，把全市人民对美好生活的新期待作为政府工作的全部奋斗目标，不断增强进取心和责任感，始终保持“既有老黄牛品格又有千里马气势”的工作状态，以踏石留印、抓铁有痕的精神，推动既定工作目标的落实。

切实加强绩效管理，科学设计政府工作流程，加强基础性工作，提高工作的专业化标准，深入研究重大问题，努力提高科学决策、民主决策、依法决策水平。坚决贯彻落实省市委重大决策部署，自觉接受人大的法律和工作监督、政协的民主监督、社会各界和人民群众的监督。

政府组成人员和工作部门，要带头严格执行中央和省市相继出台的一系列反对“四风”、廉洁自律、厉行节约等规章制度，继续加大行政监察、审计监督、行政问责力度。今年，政府性楼堂馆所一律不得新建，财政供养人员只减不增，“三公经费”和一般性支出同比分别压缩15%，把更多财力用于保障和改善民生，在政府自觉过“紧日子”中，让群众过上“好日子”！

各位代表，今年，我市改革发展、改善民生的任务十分艰巨。让我们在市委的坚强领导下，锐意进取，真抓实干，朝着既定的目标砥砺前行，用实干成就伊春转型跨越、绿色崛起的伟大梦想！
